# Obituary

**Published:** 1883-06

**Authors:** 


### Obituary.

Died, in Fort Wayne, Ind., on the 9th of May, 1883, ISAAC KNAPP, M.D., D.D.S., aged 69 years.

Dr. Knapp was born at Dummerston, Vermont, March 22, 1814. His literary education was completed at Marietta College, Ohio, in 1841. He adopted his father's profession, that of Medicine, and graduated at the Medical Department of the Vermont University, and practiced for many years at Pomeroy, Ohio. His health proving inadequate to the labors of a general practice, he prepared for the specialty of Dentistry, and commenced its practice at Fort Wayne, Ind., in 1855.

He was thrice chosen President of the Indiana State Dental Association, and held many positions of honor in the foremost

Dental Societies of this country. Dr. Knapp was a dentist of phenomenal ability, as the beauty and perfection of his work stand to verify. Always courteous and considerate to his co-laborers in the same field, all were refreshed and encouraged by contact with him. The dental profession in general and in Fort Wayne in particular can with earnestness and truth say *their* professional standard has been elevated by his precepts and example. They will cherish his memory for the honor and character he has made for those who follow him. S. B. B.

